# Baron Larrey's Chirurgical Report of the Events of July 1830, in the Military Hospital of Gros Caillou

**Published:** 1832-01-01

**Authors:** 


Relation Chirurgicale des Evénements de Juillet 1830, a l'Hôpital Militaire du Gros-Caillou. Par *Hippolyte Larrey*, Chirurgien-sous-Aide-Major. Second Edition; à Paris, 1831.

A sanguinary contest of three days, in the heart of a large city, amply provided with extensive hospitals, attended by surgeons of the highest reputation, who were enabled to give up their whole time and attention to the wounded, and to perform the necessary operations under the most advantageous circumstances, is an event in the history of modern military surgery of the highest importance. The military surgeon, in general, has to perform his duties under very different circumstances; he operates on a field of battle, or in a hastily-constructed hospital; his patients probably are subjected to immediate removal on badly-suspended vehicles; or he is obliged to confide their after-treatment to other and less skilful hands; the wounded are among strangers in a foreign land; and the consequent mental depression increases the chances of unfavourable result. But in Paris, in July 1830, the wounded were immediately distributed to the different hospitals which were almost emptied for their reception; the surgeons and assistants were in constant attendance, and the cases were daily watched by them until their termination. Subsequently the principal hospital surgeons have made reports to the Institute of their successes or failures, and a better opportunity perhaps was never offered, of judging of the comparative results of the means employed, from all the patients, as far as relates to attendance and comforts, having had an equal chance of success. M. Dupuytren delivered in his course of clinical lectures a particular and daily account of all the important cases submitted to his care at the Hôtel Dieu, as well as a series of lectures on gun-shot wounds. These will shortly be published by Messrs. Marx and Palliard, under the Baron's immediate inspection. Baron Larrey, the surgeon-in-chief of the Military Hospital of the Ex-royal Guard, (Hôpital Militaire du Gros Caillou,) presented a short report to the Institute of his cases, a few months after the revolution of July; and his son, M. Hippolyte Larrey, one of the most promising young surgeons in Paris, has, in the work which heads this article, extended the report given by his father, and rectified and completed that which was incorrect or imperfect from the haste in which it was written. M. Dupuytren was directed by the Institute to examine the work; and his report bears the most honorable testimony to the zeal, intelligence, and information of the author.

M. H. Larrey was attached as an army surgeon to his father's hospital, and he fully availed himself of the opportunities he possessed of taking accurate notes of all the cases admitted.

Two hundred and sixty-six soldiers of the ex-guard, wounded in the three days, were received into the HÔPITAL DU GROS CAILLOU, of which twelve were afterwards transferred to the civil hospitals, and three to private residences. Of the remainder, four were brought in dead, four dying, twelve died subsequently, and 231 were cured.

The soldiers were not in general wounded, as in battle, by cannon or musket balls; the greater number of wounds were inflicted by slugs; musket-balls were cut into "lingots" to fit fowling-pieces, and, as in the insurrection at Cairo, children's marbles were substituted for bullets, and produced more serious injuries—many wounds were inflicted with small shot. The citizen-soldiers did not employ fire-arms alone; every species of furniture, logs of wood, tiles, and especially paving stones, (each of which weighed at least 50lb.) were hurled from the windows on the heads of the regular troops. The greater number of wounds were produced by fire-arms discharged from a short distance; and they were consequently much more dangerous than those received in battle. The flying surgical carriages (ambulantes volantes) which were first employed by Baron Larrey in Buonaparte's first campaign in Italy, and the importance of which is so fully discussed in the "Memoires de Chirurgie Militaire," were stationed near the fighting troops, so that the wounds were simply dressed previous to the soldiers being conveyed to the hospital.

M. H. Larrey has successively described the wounds of the head, chest, abdomen, and limbs, prefacing each division with a general description of the treatment employed by his father. The practice of enlarging gun-shot wounds, except for the purpose of facilitating the extraction of splinters of bone and foreign bodies, or the application of ligatures to bleeding vessels, is, as Dr. Hunter has stated, rarely employed by British army surgeons. John Hunter discountenanced it, as he considered that it tended to increase the inflammation and retard the healing of the wound. The experience of Baron Larrey leads him to a directly contrary conclusion; he directs the enlargement of almost all gun-shot wounds, especially of those which come under the following heads.

1. Superficial wounds with ecchymosis.

2. Superficial wounds with destruction of the integuments.

3. Deep wounds without primitive complication, but which will probably be followed by severe inflammation or nervous irritation.

4. Injury of the nerves, which may be followed by tetanus.

5. Gun-shot wounds tearing away tendons and muscles, followed by inflammatory congestion, nervous symptoms, purulent effusion, sanguineous infiltration, and sphacelus.

6. Wounds with primitive, or consecutive hæmorrhages.

7. Wounds complicated with fractures.

8. Wounds containing foreign bodies, such as projectiles, splinters of bones, &c.

In many cases of this kind, a simple enlargement of the wound is much more safe than rude attempts at the extraction of the foreign body by means of forceps, bullet extractors, or other instruments. The wounds having been enlarged, their edges are to be brought accurately together, and maintained in apposition by dressings; these are to be slightly tonic and compressive. The dressings are simply the unguentum styracis spread on linen pierced with numerous holes, (linge penêtré,) charpie, compresses steeped in cold camphorated vinegar, and a roller producing a regular compression. This mode of dressing generally succeeds so well, that subsequent inflammation seldom requires the employment of antiphlogistic remedies; but, in many cases, general bleeding is practised at first, rather as a measure of caution, than of necessity—in some cases, on the contrary, where there is stupor from the violence of the shock, or syncope from hæmorrhage, bleeding is entirely contra-indicated. Baron Larrey rarely employs leeches; he has observed that they favour sanguineous congestion instead of diminishing it, require the removal of the dressings which should be seldom changed, and do not furnish a sufficient quantity of blood unless they are applied in such numbers and so frequently, that the length of the process prevents the dressing of the wound, the necessary operations, and the repose of the patient. Occasionally the bites of the leeches are so deep that small vessels are wounded, the hæmorrhage from which is with difficulty arrested. The leech-bites suppurate and ulcerate, and frequently the division of small nervous filaments occasion symptoms attributed to other causes. M. Larrey abstracts blood locally by means of cupping-glasses; instead of the German scarificator employed in this country, and to which he objects from the shortness and depth of its incisions, and the difficulty of directing them, he lightly scarifies the skin with a razor, not penetrating deeper than the cutis, and in a direction parallel to the nervous filaments of the skin. One of the chief peculiarities in his practice is the length of time that he keeps the same dressing applied. In simple wounds he does not renew it until from the 4th to the 8th day; in complicated wounds without fracture, a little sooner; but on the contrary, later in wounds complicated with fractures; in these, as well as in cases of simple fracture, the first dressings are not removed until from the 40th to the 60th day, according to the severity of the fracture. The longer the time the fracture will require to consolidate, the longer are the first dressings to remain applied. The principal advantages of this method are—

1. To avoid the frequent contact of the air with the wounds.

2. To diminish greatly the abundance of the suppuration which is so often attended with unfavourable results.

3. To maintain the parts in a state of apposition.

4. To enable the surgeon to pay greater attention to the rest of the wounded, if their number is considerable, as happened in July, and as is generally the case in the army.

5. To allow the removal of the patient to a distant place without the wound requiring any dressing during the journey.

The efficacy of this treatment of compound fractures, which is so diametrically opposed to that enforced by Pott in this country, and generally adopted by English surgeons, namely, of dressing a compound fracture every 24 hours, is illustrated by two remarkable cases. In one of these, (Case 17,) a musket-ball produced a compound and comminuted fracture of the upper part of the humerus, splinters of bone were extracted equal in bulk to one inch and a half of the cylinder of the bone, the dressings were not removed until 25 days after the wound had been received, and then only on account of the distressing itching produced by maggots; the wound was found in a healthy state, and the cure was rapid. In the second case the upper part of the thigh-bone, immediately below the trochanter minor, had been fractured into several splinters, many of which were extracted; the apparatus was removed 50 days after the injury to satisfy the curiosity of some foreign surgeons who wished to see the state of the wound, which was found to be in a most favourable condition, (Case 23.) In examining the medical practice of other nations, we do not perhaps take sufficiently into account the differences of constitution produced by the climate, diet, and habits of the people; it is evident that if the treatment which Baron Larrey recommends were adopted in the London hospitals in cases of compound fracture of the limbs of young and healthy men, that it would be followed by severe inflammation, and its worst consequences; but in Paris, where, instead of large quantities of malt liquor, deleteriously drugged spirits, and much animal food, the diet of the poorer classes consists of light acid wines, much vegetable, and but little animal food, and purer and weaker spirits, the chances of inflammation are considerably less, and smaller doses of medicine act with greater power. The pay of the French soldiers is very small, four sous per diem is all that remains after the expenses of the mess, &c are paid; the greater number are thus precluded from indulging in intemperate habits: it will be seen by the subsequent cases that the wounds of the officers, who are not thus circumstanced, were in general followed by more serious inflammation. Some difference also may be attributed to the rigorous attention to diet, so universally prevalent in the hospitals of Paris, and which is owing to the remarkable influence of M. Broussais' doctrines, which attribute to "gastro-enterite" "all the evils which flesh is heir to."

### WOUNDS OF THE HEAD.

In lacerated wounds of the scalp Baron Larrey pares the edges, brings them together with adhesive plaster, covers it with linen spread with "ung. styracis," or with charpie or compresses steeped in cold camphorated vinegar, and surrounds the whole with a roller; the dressings are not to be removed before the 6th or 8th day. The bandage must be lightly applied, as considerable compression produces erysipelas of the scalp, and nervous or inflammatory affections of the brain itself, which may require the application of ice to the head, bleeding from the temporal artery or jugular vein, cupping from the nape of the neck, epigastrium, or hypochondrium, cooling drinks, and in a few cases slight purgatives.

*Case* 1. T.——, brigadier of the ex-guard, was struck on the 28th July with a paving stone on the head, the shock of which was so violent as to throw him from his horse; the helmet had but slightly warded off the violence of the projectile, which had produced a wound on the sinciput, on a level with the sagittal suture, two inches in length; the scalp was destroyed at that part, but no fracture of the denuded cranium could be discovered; the wound was simply dressed at the time, but the man was not sent into the hospital until the 3d of August. On his admission he was in a lethargic state. The wound began to cicatrize favourably, and he was about to leave the hospital, when on the night of the 6th day he was awoke by a sudden and profuse hæmorrhage. M. H. Larrey removed the dressings, and found that the blood proceeded in a double jet from an angle of the half-cicatrized wound; he applied a ligature to a small artery of the scalp, and as this did not arrest the bleeding, he applied a compress and roller; this restrained it until the morning, when it re-commenced on removing the dressings. Baron Larrey then enlarged the wound, tied an artery, and re-applied the dressings; five days after, (Aug. 14th,) the hæmorrhage recurred with alarming symptoms, such as debility, coma, and erysipelas of the scalp, spreading to the shoulders. The wound was again enlarged by a crucial incision, which, by exposing a larger extent of the cranium, proved that there was no fracture; the deep arteries of the pericranium were discovered to be the source of the hæmorrhage. The actual cautery was then applied to the bottom of the wound, to its edges, as well as to the whole erysipelatous surface; this, together with ice on the head, and cupping the epigastrium, cured the erysipelas, but the bleeding occurred a fourth, fifth, and sixth time, whilst the general debility increased. Another application of the red-hot iron, ice on the head, slightly compressive dressings, elevated position, complete rest, and a severe diet, consisting only of cooling drinks, at last effectually prevented the return of the bleeding. The man left the hospital again in September to re-enter the army. We can hardly agree with M. H. Larrey that this is a case which demonstrates "les heureux effets qu'on peut obtenir du cautère actuel" in hæmorrhage; the bleeding recurred three times after the first application, and the second trial was accompanied by means which, in most cases, are alone found capable of arresting the severe hæmorrhage.

*Case* 2. P——, artillery-man of the ex-guard, was wounded near the right ear, July 29th; two slugs had apparently entered together on a level with the mastoid process, and had passed out, making two holes, one through the antihelix, the other more anteriorly, on a level with the zygoma. The base of the mastoid process was splintered, and the squamous portion of the temporal bone fractured. There were symptoms of violent concussion and compression of the brain. Baron Larrey immediately enlarged the wound, and extracted the splintered fragments of bone; one of these was the size of the crown of a small trepan; its removal gave exit to some effused blood, with great relief to the patient; by placing the finger on the exposed dura mater, the pulsations of the brain could be felt. A seton was passed from one wound to the other, to facilitate the discharge of pus; simple dressings were applied to the wound—ice to the head. The symptoms of compression were sensibly diminished, but the intellectual faculties were considerably disturbed; the right ear was deaf; questions were answered incoherently, and the extremities were benumbed. Cupping on the nape of the neck, between the shoulders, and on the epigastrium—general bleeding, emollient poultices, ice constantly on the head, and cooling drinks, had much alleviated these symptoms, when, on walking across the ward to the watercloset, September 1st, the patient felt symptoms of apoplexy come on, which speedily terminated his existence.

*Examination of the Body 24 Hours after Death.* Effusion of bloody pus in the superior longitudinal sinus; rupture of the membranous parietes of the right lateral sinus; injection of the cerebral veins; sero-sanguineous effusion in the lateral ventricles, on the surface of the cerebellum, and in the commencement of the spinal canal. Pleuro-pneumonia of the left side of the chest, with adhesions and effusion. This alteration, which was evidently chronic, had manifested itself since the wound only by slight cough and dyspnœa. The preceding year, the patient had been treated at the hospital for acute pneumonia.

*Case* 3. The particulars of this case, which was not placed under the care of the military surgeons, were given by M. Magistel. M ——, a locksmith, believing that he was followed by his companions, rushed alone into the middle of a troop of horse-soldiers. He fell immediately, having received seven or eight sabre-wounds of the head. He was carried into a neighbouring house, and the wounds were dressed. One of the blows had removed a portion of the right parietal bone, two inches in length and one in breadth, so that the dura mater was exposed, the flap of integument was turned downwards over the ear; a second flap, on the superior part of the head, having its base inferiorly, was almost detached from the integuments; a third, extending behind and above the left ear, and laying bare a considerable portion of the cranium, exposed a transverse furrow in the bone, two inches in length and two lines in breadth; the dura mater was exposed, but not injured. The man was agitated, and talked incoherently; his pulse very weak; he drank water frequently, and had one stool. M. Magistel tied a small artery of one of the flaps, removed the splinters of bone from the dura mater and the internal surface of the integuments, united by three points of suture the superior flap, and brought the others together by adhesive plaster. The patient was carried to the Hôpital Beaujon, and placed under the care of M.M. Marjolin and Blandin. Slight inflammation of the brain yielded in a few days to proper treatment, an abundant hæmorrhage was with difficulty arrested, and a small collection of pus which formed above the dura mater was evacuated; on the 24th of August he began to take food, and, at the beginning of September, he was removed to the "Maison de Convalescence" at Saint Cloud, a building appointed by the Government for the reception of the convalescent wounded. This patient was introduced at one of the sittings of the Royal Academy of Medicine of Paris.

### WOUNDS OF THE FACE.

When they are deep, extensive, and irregular, Baron Larrey enlarges them according to their situation; he pares the edges until they are perfectly linear, and unites them by suture. The loose, extensible, and elastic tissues of the face readily admit the traction of ligatures; from the same causes inflammation is with difficulty excited, and a regular and hardly perceptible cicatrix is the result of an extensive, deep, and deforming wound. The Baron agrees with Dessault in recommending the twisted and quilled suture, so as to act gradually on the whole thickness of the edges of the wound.

*Wounds of the Eyes.*—*Case* 4. M. D——, captain of the 50th regiment of foot, lost the sight of the right eye by a small shot penetrating the cornea, iris, and hyaloid membrane, without the evacuation of the humours, or loss of the natural form of the organ. It was followed by slight symptoms of inflammation of the brain, which were cured by moderate antiphlogistic treatment.

*Case* 5. The left eye of M. J——, lieutenant of the 7th regiment, was entirely disorganized by a gun-shot wound, without any injury of the eyelids. The consequences of the wound were alarming, from an erysipelas, which successively affected the left and right side of the head and the neck. At the commencement, it put on a phlegmonous character, the pain increased, the traumatic fever was constant, with exacerbations on the least excitement, and extreme general anxiety. The patient's life was saved by the application of the actual cautery many times to the whole erysipelatous surface, seconded by antiphlogistics.

*Case* 6. P——, soldier of the 6th regiment, received a ball in the left orbit, which entirely destroyed the eye, and fractured the external wall of the orbit, without implicating the eyelids; their external angle only was torn, but it cicatrized regularly. No unfavourable symptoms came on.

*Fractures of the Maxillary Bones.* The most dangerous gun-shot wounds of the face, are the comminuted and compound fractures of the jaws. The treatment employed by Baron Larrey consists in enlarging the angles of the wounds, in extracting the moveable splinters, and, as far as is possible, all foreign bodies, in removing the irregular edges of the wound, or disorganized flaps of integument, in bringing the lips together, and maintaining them in contact by suture, and in applying dressings which are not to be removed before the 8th, 10th, or 12th day.

*Case* 7. M. M——, captain of the 7th Swiss regiment, aged 56, was struck on the right side of the lower jaw by a mass of iron, thrown from a distance of ten paces. He was immediately carried to the hospital. The aspect of the wound was hideous; the face covered with blood; the mouth extended and strongly retracted downwards, so as to expose the whole extent of the wound. The cheek was torn through its whole thickness, the external jugular vein and many cervical nerves were divided, and nearly the whole of the body of the inferior maxillary bone was fractured in splinters. The poor officer, who was covered with twenty scars, received in the campaigns of Napoleon, was less alarmed than the surgeons. M. Larrey commenced by paring the edges of the wound, and extracting all the moveable splinters of bone, one of which was two inches in length; he then tied a branch of the facial artery, brought together the edges of the wound, and kept them in contact by many points of suture and a bandage. The case went on favourably, the wound beginning to cicatrize by the 15th August. After this period, the cure was delayed by untoward symptoms. Secondary splinters were obliged to be extracted, abscesses to be opened, points of suture to be renewed, and the actual cautery to be applied for an extensive traumatic erysipelas; the suppuration was abundant and tedious. Notwithstanding these hindrances, the wound was completely cicatrized by the month of October, and an exact and linear union was the result of the hideous wound. The inferior jaw was reduced in size, from the quantity of splinters of bone removed immediately after the injury, but this loss did not inconvenience the action of the mouth.

*Case* 8. M——, upwards of 50 years of age, pioneer of the 3d regiment of the ex-guard, was struck, in the morning of the 29th of July, with a ball, which fractured his jaws. The following were the appearances of the wound on his entrance into the hospital immediately after. The ball had perforated the right side of the upper lip, a circular portion of which, equal in size to the projectile, that is, about 14 lines in diameter, had been carried away, but without destroying the edges of the lip; the posterior half of the upper right maxillary bone and the right angle of the lower jaw were fractured, some branches of the internal and external maxillary arteries, as well as the anterior branches of the first cervical nerves, being at the same time divided. The ball was stopped by, and imbedded in, the mastoid process. The state of stupor and the symptoms of cerebral concussion, together with the appearance of the local injury, were so alarming, that, at first sight, the man appeared almost beyond the resources of art. Baron Larrey, instead of completing the division of the lip, and endeavouring to produce a linear union of the wound, which would have reduced the size of the mouth, and inconvenienced its subsequent motion, lightly removed the inequalities of the edges of the wound, brought together the borders circularly, and maintained them in contact by means of the interrupted suture. He then proceeded to remove the ball and the splintered bone. For this purpose he made an incision through the sterno-cleido-mastoideus muscle, close to its superior attachment, and seized, without difficulty, the ball; at the same time, many fragments of the maxillary bones were removed—one of them, from the ramus of the lower jaw, was two inches and a half in length; two vessels were tied. The dressing was not removed before the 7th day, and subsequently as seldom as possible. The cure was effected without any unfavourable symptom.

### WOUND OF THE NECK.

*Case* 9. M ——, soldier of the 7th regiment, was struck by a ball discharged immediately behind him. It passed through the thickness of the extensor muscles of the neck, shattered the left tranverse processes of the second and third cervical vertebræ, but without injuring the vertebral artery, and came out between the sterno-cleido-mastoideus muscle, and the cervical vessels and nerves of the same side. Paralysis of the left arm was the consequence, and tetanus was feared. The application of moxas, with cupping between the shoulders, removed these symptoms, and the cure speedily ensued. This was the only wound in the neck received in the Hôpital du Gros Caillou worth recording; and it is remarkable, from the ball having passed so near to so many important parts without injuring them. The mobility of the vessels of the neck, and the ease with which they yield to pressure; explain the infrequency of their being wounded, even by cutting instruments, in the most desperate attempts at suicide.

### Wounds of the Chest.

On referring to the tables of mortality of the different hospitals of Paris, civil as well as military, and to the examinations of bodies brought to the "Morgue," it has been found that the most common as well as the most fatal, wounds are those of the chest. It is remarkable that, out of 15 penetrating wounds of the chest which were brought into the Hôpital du Gros Caillou during "the three days," of which eight were attended with serious symptoms, but one terminated fatally, and that case was complicated with another injury, which required the amputation of the arm at the shoulder-joint. The general treatment employed was the following, which has been found by M. Larrey to be the most effectual.

1. Enlargement of the wounds in proportion to their extent and depth, in order to render them linear and more susceptible of cicatrization, to unload the vessels of the part, to prevent nervous and inflammatory complications, and to facilitate the extraction of foreign bodies.
2. The immediate union of the wounds, to preserve them from the contact of the air, to prevent emphysema, and, as much as possible, the effusion of pus in the thorax.
3. Simple dressings, seldom removed.
4. General bleeding, and local "revulsive" abstraction of blood by cupping, according to circumstances.
5. Very spare diet, elevated position of the chest, absolute rest.

*Case* 10. M——, soldier of the 1st regiment, was severely wounded by his own musket, which he had just given up to a cowardly opponent. The ball, discharged close to his body, passed through the left shoulder, fractured the clavicle, injured the brachial plexus of nerves, penetrated the chest, and, traversing the lung, came out between the third and fourth ribs, near the posterior border of the scapula of the same side. When brought to the hospital, the man was threatened with immediate suffocation, from effusion of blood into the chest; he was in a state of collapse. M. Larrey enlarged the wounds, and gave issue to some blood; he united them, and endeavoured to produce absorption of the effusion by cupping the whole surface of the chest. By these means the symptoms of suffocation were diminished; but, about the third day, those of effusion increased—the cough and dyspnœa grew more violent; the patient sought relief by lying on the left side, or by sitting up in bed; the wounded side of the chest was evidently dilated; the ribs were elevated and separated from each other; change of position produced within the thorax the shock of a liquid, which was felt by the patient himself. M. Larrey was about to perform the operation for empyema, when a violent fit of coughing produced a copious expectoration of black blood, mixed with sero-purulent fluid. This was attended with great relief, and, by seconding the efforts of Nature, and promoting expectoration, these symptoms disappeared. Owing to the injury of the left brachial plexus, the arm of that side had completely lost its power of motion, whilst its sensibility was greatly increased; by the repeated application of moxas the motions of the arm were completely restored. He left in October cured.

Seven other cases are detailed in which the chest was penetrated by balls; the general symptoms were similar to the last, but less severe; they all terminated favourably, the greater number in 30 or 40 days. In two of the cases the balls apparently remained in the chest; in one of them it was probably fixed between the arches of two of the ribs, as towards the inferior border of the serratus magnus there was a fixed pain, augmented on pressure. M. H. Larrey remarks, that care should be taken in not deciding too positively in these cases on the lesion of the lungs themselves, as from the well known deviation of balls, the diagnosis should be formed from the nature of the symptoms rather than from the external situation of the wounds; a ball passing into the cavity of the chest may, instead of following the direct line of impulsion, take the course of the curvature of the ribs, and even pass out at the same side of the chest that it had entered. The general symptoms in all these cases prove satisfactorily that the lungs themselves were wounded.

### Wounds of the Abdomen.

In cases of penetrating gun-shot wounds implicating the abdominal parietes alone, M. Larrey recommends the careful enlargement of the wound in the direction of the muscular fibres, and prefers position with a suitable bandage to gastroraphy; he considers that the stitches of sutures, however near together, do not always prevent herniæ, from which succeed, irritation, inflammation, strangulation of the gut, rupture of the integuments which have been sewn, traumatic fever, and sometimes peritonitis, gangrene, and death. He believes that wounds of the intestines are much more susceptible of cure than is generally supposed, and on the principle, that nature in such cases does more than art, he does not attempt to find the wounded gut; he enlarges the wound, dresses it simply, puts the patient on the spare diet, employs general and local bleeding, oily embrocations over the belly, tepid baths, emollient glysters, cold mucilaginous drinks, and sometimes a mild laxative. He divides the curative process of wounded intestines into four periods. In the first an eschar forms; in the second it is detached and gives exit to feculent and purulent matter, which continues to escape for a time, longer or shorter according to circumstances; during the third period this discharge generally diminishes in the same proportion, until it ceases to be poured out externally; the union of the wound constitutes the fourth period, the corresponding textures coming into contact cicatrize from within outwards; the primitive adhesions become absorbed, and finally there remains only a slight contraction of the intestinal canal at the wounded part.

*Case* 2. B——, soldier of the 1st regiment, received in the left flank a ball, which, passing from before backwards, penetrated beneath the last cartilage of the false ribs, traversed the peritoneal cavity, perforated the sigmoid flexure of the colon, and came out at the lumbar region of the same side. During two days the liquids taken into the stomach flowed with pus through the abdominal wound; as the purulent matter became more abundant, the escape of the contents of the intestine subsided: this was followed by an attack of acute peritonitis which yielded to the treatment mentioned above. At the commencement of September the wounds had healed.

Count Belliarde, one of the most distinguished generals in the expedition to Egypt, was wounded in the same manner at the revolt of Cairo, and was treated by Baron Larrey with the same success.

*Case* 12. B——, soldier of the 6th regiment, was wounded by an iron bar which is used at the Custom Houses to search bales of goods. This was driven into his right flank, and perforated the ascending colon; the lavements and fæces escaped from the wound for nine days. The general and local symptoms were severe, but gave way under antiphlogistic treatment, and the patient left the hospital cured.

*Wound of the Bladder. Case* 13. J——, a Swiss soldier of the 7th regiment, was struck on July 28th by a ball, which entered the right groin and made its exit at the left side of the nates. The direction of the wound, and the immediate symptoms, proved that the bladder and the rectum had been wounded; the urine escaped from the rectum and from the two wounds. Baron Larrey enlarged the wounds, simply dressed them with cerate spread on linen, charpie, compresses, and a bandage, and fixed in the urethra a gum-elastic catheter. For the first few days there were inflammatory symptoms about the abdomen, acute pain, fever, want of sleep, and diarrhœa; general bleeding, oily embrocations over the abdomen, emollient poultices, clysters (half a pint), cooling mucilaginous drinks, perfect rest and suitable position, spare diet, great cleanliness, and, in this case, the frequent renewal of the dressings subdued the inflammation. During the first ten days the urine mixed with pus escaped by the two wounds, as well as by the rectum and urethra, after that time it gradually résumed its natural course. The 29th of August, a month after the receipt of the injury, the flow of urine from the anus was reduced to a simple exudation, and the wounds were almost cicatrized. Baron Larrey, in his report at that time delivered to the Academy, anticipated the ultimate cure of the patient—this, however, was unfortunately not realized. Experiencing slight inconvenience from the constant presence of the catheter the patient removed it, and in endeavouring to replace it himself, he destroyed a small portion of the mucous lining of the urethra at the bulb; the consequences of this was a urinary fistula, and a return of all the general and local inflammatory symptoms; the wounds became deep and livid, the suppuration profuse, the strength of the patient daily more exhausted, a large slough separating laid bare the sacrum, the wounds became gangrenous, and acute peritonitis terminated his life towards the end of October.

*Examination of the Body* 24 *hours after Death.* Gangrenous state of the wound in the groin; effusion of urine into the cellular tissue of the pelvis; destruction of about an inch of the middle part of the right side of the bladder, which viscus was much thickened, contracted, and inflamed. The wound of the rectum was not apparent; the posterior wound was partly cicatrized. The bladder contained a few splinters of bone from the fractured os pubis. The peritoneum bore the marks of severe and extensive inflammation.

### Wounds of the Extremities.

The propriety of *immediate* amputation which has constantly been supported by Baron Larrey, is farther confirmed here. Forty cases of wounds of the extremities were received into the Hôpital du Gros Caillou, and twenty amputations were performed, seven of which were unsuccessful; of these seven, but one was an immediate amputation—the other six were consecutive.

*Case* 14. D——, a soldier of the 5th regiment, was struck by a ball, which entered near the posterior border of the right scapula, fractured that bone transversely, and passed through the thickness of the deltoid muscle. At first there were no alarming symptoms, the wound appeared simple, and was treated as such; but soon the track of the ball became excessively painful without any apparent cause, suppuration ceased, the edges of the two wounds became pulpy, brownish, and almost dry, deep lancinating pains extended through the breast and back, and were increased on the slightest pressure, with violent spasms of the whole muscular system, and opisthotonos. The tetanic symptoms were well marked before Baron Larrey saw the patient; he divided the wounds extensively, extracted some splinters of the scapula, and took blood largely by cupping; the veins of the arm were opened several times, emollient poultices, baths, fomentations, antispasmodic and opiate drinks, were tried, but in vain; in 20 hours the man died. On examination of the body, the limbs were found to be firmly contracted, and the jaws locked together. The two edges of the irregularly fractured scapulæ were teethed like a saw, many bony splinters were driven into the muscles, the nerves proceeding from the spine to the wound, as well as the ganglions near their origin, were of a deep red colour; effusion of blood had taken place into the whole length of the spinal canal between the two layers of the arachnoid membrane, and the medullary substance of the cord was infiltrated with blood. The brain was not examined. No particular alteration was remarked in the other viscera.

This was the only case of tetanus, occurring from wounds received in July, observed in this hospital; it arose most probably from the irritation of the unremoved spiculæ of bone, and M. H. Larrey thinks that it might not have been fatal had his father adopted his revulsive mode of treatment sooner. Baron Larrey relies chiefly on revulsives; he applies moxas and cupping-glasses along the whole course of the spine, makes incisions, and applies the actual cautery to the bottom edges of the wound; he then encourages suppuration from the wound, and cutaneous perspiration by frequent embrocations of hot camphorated oil of camomile. One of the most remarkable proofs of the efficacy of this decisive treatment is given in his "Clinique Chirurgicale;" it is the case of a grenadier of the Imperial Guard of Napoleon, who had the top of his shoulder carried away by a cannon ball, complete opisthotonos came on with surprising rapidity—yet he recovered.

*Case* 15. L——, corporal of the 6th regiment, was struck by a ball, which passed through the belly of the biceps muscle; the wound went on favourably; three weeks afterwards phlegmonous erysipelas commenced in the shoulder, and successively attacked the arm, fore-arm, and hand, without affecting the immediate circumference of the wound; it was attended with severe symptomatic fever and delirium; the red-hot iron applied to the whole erysipelatous surface destroyed the inflammation, and the patient was cured.

*Case* 16. M———, soldier of the 3d regiment, was wounded on the left arm by a ball discharged close to him. It passed from before backwards, fracturing into splinters the whole of the thickness of the os humeri at its upper part as far as its neck; the artery and nerves were uninjured. Baron Larrey resolved to attempt saving the arm; he enlarged both wounds considerably, and extracted numerous splinters of bone, the whole of which equalled in bulk one inch and a half of the humerus. The arm was deprived of all strength and power of motion. The wounds were dressed with many compresses steeped in camphorated vinegar; a single pasteboard splint was applied on the inner side of the arm, and a large triangular pad (the base corresponding to the elbow, and the apex fitting into the axilla, so as to separate the arm from the chest) was secured by a large roller; the fore-arm was placed in a sling in a state of semi-flexion. This dressing remained on the arm until the 25th day after the receipt of the injury, no nervous nor inflammatory symptom having intervened; at that time, on account of a troublesome itching about the shoulder, the dressing was removed; this was found to be occasioned by a multitude of maggots in the wound; they were removed and compresses dipped in a camphorated liquid were applied to prevent their return. The dressing was carefully re-applied, and, at the end of August, it had been changed but three times; the arm had then in part regained its strength, and the subsequent dressings were more frequent, and at last daily, until the complete consolidation of the bone and cicatrization of the wound had taken place, which was in October, when the patient left the hospital. The only inconvenience resulting from the injury was a shortening of the bone to the extent of an inch and a few lines, corresponding to the substance of bone removed. This case illustrates most favourably Baron Larrey's peculiar treatment; in little more than ten weeks a complete cure of a compound comminuted fracture of the humerus, with a loss of one inch and a half of the cylinder of the bone, was effected; the two wounds were considerably enlarged, and the original dressing was not removed for 25 days after the receipt of the injury, although occurring in the hottest month of the year. M. Larrey has observed, during the campaign in Syria, that the presence of maggots in wounds is rather favourable than injurious; that they seem greedy of the putrid and gangrenous parts; thus hastening the separation of sloughs, whilst they avoid the living parts. If this obervation be correct, these animals may be regarded as so many artificial absorbents, and we may expect, in process of time, to see the surgeon ordering the application of some hundred maggots, as gravely as he now applies some dozen leeches.

In two cases Baron Larrey removed the arm at the shoulder-joint. His mode of operating is as follows:—The patient is seated on a stool; an assistant is directed to raise the flaps and to compress the axillary artery at the moment the limb is being completely separated from the body. The operator commences by making a longitudinal incision from the acromion to a point an inch below the neck of the humerus, thus dividing the deltoid muscle into two equal parts, out of which he makes two flaps, an anterior by cutting from above downwards, and a posterior from within outwards. The assistant raises the flaps, and compresses the divided circumflex arteries; the articulation is thus exposed; the operator by a circular sweep of the knife divides the capsular ligament, he then turns slightly outwards the head of the bone, and as soon as he is satisfied that the axillary vessels are firmly compressed, he separates the limb from the body by carrying his knife backwards and inwards on a level with the inferior angles of the two flaps, and before the fingers of the assistant. The artery is then tied whilst compressed, so that all hæmorrhage is prevented.

*Case* 17. J——, soldier of the 6th regiment, was struck by a ball discharged close to his body, which fractured the right os humeri, and traversed the chest. The upper extremity of the humerus was fractured in splinters an inch from its neck, the ball then penetrated the chest a little above the right mamma, and passed out between the 5th and 6th left ribs, traversing the right lung and anterior mediastinum, fracturing a rib and denuding the pericardium for an extent of half an inch at the apex of the heart, so that the pulsations could be felt through the wound. M. Larrey enlarged the wounds and extracted portions of bone equal in bulk to three inches of the cylinder of the humerus. He dressed it similarly to Case 17, having previously enlarged and dressed the wounds of the chest. Notwithstanding the dangerous nature of the injury there were no severe primitive symptoms. On the third day the suppuration of the arm was so considerable that it had soaked the whole dressing, and an intense itching required its removal. A large quantity of maggots had collected; the suppuration was abundant, unhealthy, and fetid; a sinus had formed extending from the wound to the elbow; as there was no chance of saving the arm it was removed at the shoulder-joint on August 7th, before the wound of the chest had occasioned any alarming symptom. Until the 13th of August every symptom was favourable although the patient was in a very feeble state. On the 13th, from taking cold during the night, the patient had a shivering fit followed by fever and diarrhœa. On the 14th the fever had assumed an adynamic type; the dyspnœa, cough, and sense of suffocation, left no doubt of the existence of effusion into the chest, which speedily terminated his life. On examination, an effusion of a pint and a half of blood was found in the right cavity of the chest, floating in a less considerable quantity of pus; the right lung was torn and remarkably compressed. There were appearances of acute pericarditis, with albuminous effusion on the surface of the heart. The wound produced by the amputation was natural.

*Case* 18. P——, æt. 21, fusileer of the 7th Swiss regiment, received, on the 29th July, a ball in the left arm, discharged close to his body. There was a simple round wound at the upper third of the arm, on a level with the attachment of the pectoralis major at the inner border of the deltoid muscle; the motions of the arm were free and executed without any pain except at the external wound. It was regarded as so slight an injury that it was not shown to the surgeon-in-chief;—the ball had not passed out. Some time elapsed without the appearance of any particular symptom, except an acute pain in the wound caused by the slightest pressure and some common inflammatory symptoms; these increasing, Baron Larrey examined the patient; he immediately saw that the wound was not so simple as had been imagined; around it there was considerable inflammatory tension causing great pain and traumatic fever; a deep fluctuation was discovered from matter probably having its seat around the upper extremity of the bone. M. L. enlarged the wound more considerably, which gave issue to much pus; he passed a seton through to favour its further escape, and ordered a general bleeding, spare diet, &c. The suppuration increased, and local suffering with nervous excitement and continued fever; the strength declined, and in short the patient was in a state of imminent danger. On again probing the wound M. Larrey discovered a sort of erosion with loss of substance in a particular point of the os humeri; no doubt remained as to the source of the suppuration or of the course to be pursued. On the 27th August, M. Larrey removed the arm at the articulation, the man, although reduced to a skeleton and in a state of extreme debility, requesting the operation. On the dissection of the limb the muscles were found to be divided by purulent sinuses which had also detached them from the bone. The ball had traversed the whole thickness of the head of the os humeri forming a cylindrical canal of many lines in diameter without any fracture of the bone. The head of the humerus was already denuded of its cartilage. The wound was dressed so as to prevent too immediate union, and it slowly granulated. Some slightly unfavourable constitutional symptoms were subdued by mild remedies. On the 37th day the patient complained of a fixed pain near the infra and spinous fossa, increasing on pressure, with slight distortion of the part. M. Larrey made a deep incision and extracted the ball. From this time granulations proceeded quickly, and at the end of 20 days the cicatrization of the stump was completed. The general health was gradually recovered. This case proves the difficulty in the diagnosis of some gun-shot wounds; it is remarkable from the simple primary appearance of the wound, the delayed attack of the unfavourable symptoms, the success of a consecutive amputation performed under inauspicious circumstances, and the singular injury of the bone by the ball which perforated it without fracturing it.

Four amputations of the arm were performed, and these tend to prove most satisfactorily the success of *immediate* amputation; two of these amputations were primary and successful, the other two were consecutive and fatal. The two primary amputations were for compound fractures of the elbow-joint, and the cases were accomplished without any unfavourable symptoms; of the other two, one was for a compound fracture of the second row of carpal bones, followed by severe phlegmonous erysipelas of the fore-arm, and in the other a musket-ball was firmly wedged in between the ulna and radius. Several cases of severe gun-shot wounds of the fore-arm, with fracture of one or both bones are reported, none of which required amputation. The following case of wound of the head is of importance from its being followed by a large symptomatic abscess near the anus.

*Case* 19. M. C——, lieutenant of the 6th Regiment, commanded a detachment posted in a house in the Rue St. Honoré; the position was forced by the people: after receiving several wounds, this officer endeavoured to save his life by jumping from a window on a roof 20 feet beneath it; as he fell he received a ball through the right hand; from being a tall and large man the shock of the fall was so violent that it produced stupor, concussion of the brain, numbness and rigidity of the limbs, especially of the left arm and of the feet. The wounds were immediately dressed; that of the hand was the only serious injury: it was found that the second metacarpal bone had been fractured, and the flexor tendons and muscles torn; the wound was enlarged, splinters of bone extracted, and a suitable dressing applied. Two days afterwards he was removed to the hospital; he was bled from the arm, cupped between the shoulders. At the beginning of September, (the violent symptoms of cerebral concussion not having completely subsided,) a vast abscess formed at the margin of the anus in the substance of the right gluteus muscle, without any known cause, or well-marked local symptom; there was neither redness nor fluctuation; the fixed pain and the fever were the only indications. Baron Larrey, convinced of the existence of an abscess, made an incision two inches in depth, from which escaped a considerable quantity of pus; he then made the incision crucial, and introduced a pledget of charpie to prevent the wound's closing. From this time the unfavourable symptoms gradually abated, and he left the hospital cured.

*Wounds of the Thigh.* These were numerous, and in general slight; three only were of a serious nature; two of these were muscular wounds, followed by phlegmonous erysipelas, and they illustrate the formidable treatment adopted by Larrey in this disease.

*Case* 20. M. de Saint-C——, chief of a battalion of the 1st Regiment, received a wound from a marble discharged from a musket in the right thigh, at its superior third. The wound was enlarged and dressed; three days after, a painful spot, two inches above the patella, indicated the presence of the marble, which was extracted by a counter opening. The whole limb was attacked with severe erysipelas, threatening to extend to the pelvis; the upper part of the leg and the knee were in a state of ecchymosis, and the thigh enormously swollen; the actual cautery was applied over the erysipelatous surface, followed by a compressive bandage; this produced the best effects, and in the beginning of September the patient left the hospital quite cured.

M. Larrey has found that wounds made by balls of marble are attended with more serious injuries than those occasioned by leaden bullets. He considers that a ball of lead, from its weight, traverses a limb more easily, causing a regular wound without seriously disorganizing the surrounding parts, and that the lightness and elasticity of a ball of marble causes deeper and more irregular wounds with much more extensive disorganization of the soft parts. The case just detailed tends to confirm his opinion. This officer had been frequently wounded in the course of a long military career, and no wound from bullets had ever been attended with such alarming symptoms.

*Case* 21. R——, soldier of the 50th Regiment was struck with a paving stone on the left thigh, which produced a severe contusion, at the same time that a ball passed through the limb; there was no fracture of the femur. The injuries were immediately followed by most severe general symptoms; delirium, traumatic fever, and great mental agitation; the whole thigh became affected with severe phlegmonous erysipelas; it was enormously swelled, very painful, and without power of motion. It was feared that the inflammation would extend to the abdomen, and that there was but little chance of saving the man's life. Baron Larrey immediately made two long and deep incisions in the thickness of the thigh, which gave issue to a large quantity of pus, and applied the actual cautery at a white heat to the edges of the wounds and to the whole erysipelatous surface, not sparing a single spot. The unfavourable symptoms were immediately relieved, and they gradually disappeared. The wounds from their extent and depth very slowly cicatrized, and required the most attentive superintendance for many months.

It will be seen from several of the previous cases that Larrey applies the actual cautery to the whole surface of the skin affected with traumatic erysipelas, both simple and phlegmonous. Cases 1, 5, 7, and 15, were those of simple erysipelas of the head, neck and shoulders, and of the thigh. Cases 20 and 21 were of phlegmonous erysipelas of the arm and thigh. The whole of these were severe examples of the disease, and the treatment was successful. In Case 21, where the thigh was attacked with phlegmonous erysipelas, as suppuration had taken place, deep and long incisions were made previous to the application of the actual cautery; M. H. Larrey remarks "on sera peut-être tenté de dire que c'est là une chirurgie effrayante; mais aussi on sera forcé de convenir que c'est une chirurgie positive, une chirurgie qui sauve la vie." The symptoms were indeed formidable, and yielded to the severe means employed; but we find that milder measures frequently are as successful, and as even army surgeons shuddered at the idea of Mr. Lawrence's long incisions, this additional severity is hardly likely to be adopted at all in this country. The argument brought against the practice of incisions was, "are all the principles of surgery now so changed, and is the nature of the human body and constitution so altered, that inflamed parts are to be soothed by maiming and wounding them?" but in these cases, both simple and phlegmonous erysipelas have been cured by a remedy which is often found to produce, not only severe inflammation of the skin itself, but also of the subjacent cellular tissue. How such a remedy acts we cannot perhaps satisfactorily determine; we see, as in cases of erysipelas treated with nitrate of silver, the effects, but are we to deny the utility of the means, because they interfere with our ideas of "the nature of the human body, and the constitution," when the laws especially regulating the changes produced by disease are so imperfectly understood?

*Case* 22. H——, soldier of the 7th regiment, was wounded by a ball, which, passing through the upper-third of the left thigh, from without inwards, fractured the femur close to the little trochanter without wounding the femoral artery. He was brought into the hospital in a state of stupor. M. Larrey enlarged the wound and extracted numerous portions of bone, one of which was two inches in length. The injury was so severe that it was a question of amputation, but M. L. resolved to endeavour to save the limb, and placed it in his accustomed immoveable apparatus. The limb remained confined in this apparatus for fifty days, at the end of that time the dressings were removed, not from necessity, but to show the limb to some foreign surgeons who wished to see the result of such treatment. The limb was perfectly straight, and there was a shortening equal to the splinters of bone removed. The apparatus was again applied, but unfortunately the patient, without the surgeon's knowledge, was transferred to another ward without due care; the ends of the bone were displaced, and never afterwards regained their straight position. The bone eventually united, but with deformity. M. Larrey generally amputates the thigh after a compound and comminuted fracture of the femur by a gun shot. If an untoward event had not disappointed the just expectations of a complete cure in this case, it would have been one of those rare instances of union without deformity after this form of accident. M. Larrey prefers the circular incision in amputating the thigh; the flap operation should only be employed in those cases in which it is indicated by the disorganization of the soft parts. Of five amputions of the thigh performed in the hospital, two were primitive and successful, the other three were consecutive; one of these was fatal. One of the two primitive amputations was from a gun-shot wound penetrating the joint, the other from a fracture of the inferior third of the femur and of the condyles; the stumps were healed by the first intention. Of the consecutive amputations two were performed successfully for gun-shot wounds of the knee-joint, simple in appearance at first, but subsequently attended with severe symptoms; one limb was removed on the tenth day, the other on the 30th day after the accident; the union of the wounds by the first intention was not attempted; in both cases the cicatrization was long and tedious, and traumatic fever and diarrhœa gave serious apprehensions as to the ultimate success. In the unsuccessful case the ball had lodged in the internal condyle of the femur; the wound, apparently superficial, was attended after some time with such serious local and general symptoms, that the limb was removed 24 days after the injury; death ensued 12 days afterwards. In the year 1818 M. Larrey had removed this patient's right testicle on account of a schirrous affection; on examining the body a marked depression was seen on the right side of the os occipitis, and the corresponding lobe of the cerebellum was less than that of the left side. This remarkably confirms the assertions of Gall on the sympathy between the testicles and cerebellum. The results of amputations of the leg were less favourable than those of the other operations, perhaps owing to the more severe and complicated injuries. Two primitive amputations were performed, one (Case 23) was fatal, the other (Case 24) was successful; there were three consecutive amputations, and but one succeeded; this was a case of compound fracture from gun-shot wound of the foot; the amputation was performed 14 days after the receipt of the wound, and the stump was healed on the 25th day. This was the only case of consecutive amputation which so completely succeeded.

*Case* 23. Count de P——, æt. 48, of a nervous temperament and spare habit, formerly an aide-de-camp of Demoustier, who by his bravery and military talent had greatly distinguished himself in Spain, Holland, and Prussia, commanded as colonel the third regiment of the royal guard on the 27th of July. Riding before the troops, and consequently being much exposed, he received, on the Boulevards, about 11 o'clock in the morning, two balls in his right leg—his horse was killed under him. The surgeons of the regiment, who were on the spot, slightly dressed his wounds; but the regiment being surrounded by the populace, without possibility of retreat, were obliged to sustain the combat until the evening; the citizens then gave way, and at the Colonel's own request he was brought to the Hospital of the Guard; owing to many obstructions in their progress they did not arrive there until midnight, twelve hours after the receipt of the injury. Baron Larrey found the whole leg cold and without sensibility. A ball had fractured the outer ankle, and another had torn the anterior tibial artery, and fractured the middle third of the tibia. This extensive injury left no doubt as to the propriety of amputation; but was it prudent to perform it immediately? The state of anxiety of M. de P. who had remained twelve hours wounded without wishing to abandon his soldiers, the shock sustained by the fall of his horse and by the fatigue of his transport to the hospital, determined Baron Larrey to defer the amputation for some hours, in order to calm the agitation of his mind, and if possible to establish the heat and vitality of the leg. A little broth and Bordeaux wine were given, the surface of the body was rubbed with dry flannel, and the limb was warmly covered. At five in the morning M. de P. was calmer, the heat of the limb had slightly returned, but sphacelus had commenced on the foot; after much hesitation on the part of the patient the limb was removed. On account of the extent of the upper wound, and the necessity of removing the limb as far from the seat of gangrene as possible, M. Larrey sawed through the head of the tibia and disarticulated the head of the fibula; from the extreme irritability of the stump, and the stretching which the skin would require in order to cover so large a surface, the edges of the wound were not brought together so as to be united by the first intention. Until the 8th day the symptoms were satisfactory; at that time a slight fever commenced, with symptomatic irritation of the stomach, and anxiety; the aspect of the stump subsequently changed, suppuration was abundant and fetid, attended with the most aggravated symptoms of severe constitutional irritation and cough; the patient became delirious, and died on the 29th of August.

*Examination of the Body 24 hours after Death.* Effusion of serum into the cranium, injection of the arachnoid membrane, "ramollissement" of the brain, considerable effusion into the chest, adhesions and false membranes, tubercles in the lungs in a state of suppuration, abscess in the liver, inflamed patches in the stomach and intestines. The knee-joint was filled with purulent matter, and the muscles of the inferior third of the thigh were infiltrated with pus, and reduced almost to a state of putridity.

*Case* 24. M. G. one of the oldest lieutenants of dragoons, was shot in the leg whilst passing with his regiment through the Champs-Elysées. His horse fell with him, his wounded limb being beneath the animal. The shock was tremendous, and the officer was brought into the hospital in a state of complete stupor and apparently dying. Slight stimulants and warm frictions were employed with good effect. Both bones of the leg had been fractured by two balls at the union of the middle with the superior third of the limb, with disorganization of the soft parts, considerable ecchymosis and inflammatory swelling; the limb was removed as high as possible (as in Case 23) two hours after the receipt of the injury. It was borne with the greatest fortitude; immediate union was attempted; blood was taken from the arm, and, by cupping, from the hypochondriac and epigastric regions, and the strictest diet was enjoined. The symptoms were favorable until the fourth day, when the knee became red and painful, and the patient grew feverish and delirious. A traumatic erysipelas attacked the whole thigh, and the stump became affected with hospital gangrene; this state was accompanied with stupor and numbness. M. Larrey applied the actual cautery to the erysipelatous surface and to the stump; he abstracted blood by cupping from the epigastric and hypochondriac regions, and applied dry cupping-glasses over the abdomen. This active treatment subdued the erysipelas, and the wound lost the characters of hospital gangrene, but the traumatic fever remained; this was discovered to arise from a large collection of pus beneath the fascia lata of the upper part of the thigh, probably the effect of a contusion; two large incisions were made, a seton was introduced, the wounds healed, and the fever ceased. The subsequent healing of the stump was slow. Incomplete anchylosis of the knee-joint was the only inconvenience that resulted from this very complicated injury.

